# A Pilot Study on Automatic Three-Dimensional Quantification of Barrett’s Esophagus for Risk Stratification and Therapy Monitoring

**DOI:** 10.1053/j.gastro.2021.05.059

**Published:** 2021-06-08

**Authors:** Sharib Ali, Adam Bailey, Stephen Ash, Maryam Haghighat, TGU Investigators, Simon J. Leedham, Xin Lu, James E. East, Jens Rittscher, Barbara Braden

**Affiliations:** 1 institute of Biomedical Engineering, Department of Engineering Science, University of Oxford, Oxford, United Kingdom; 2 Translational Gastroenterology Unit, Experimental Medicine Division, Nuffield Department of Medicine, John Radcliffe Hospital, University of Oxford, Oxford, United Kingdom; 3 Oxford National Institute for Health Research Biomedical Research Centre, Oxford, United Kingdom; 4 Intestinal Stem Cell Biology Laboratory, Wellcome Trust Centre Human Genetics, University of Oxford, Oxford, United Kingdom; 5 Ludwig Institute for Cancer Research, University of Oxford, Oxford, United Kingdom; 6 Big Data Institute, University of Oxford, Li Ka Shing Centre for Health Information and Discovery, Oxford, United Kingdom

**Keywords:** Imaging, Deep learning, Three-dimensional, Risk assessment, Esophageal cancer

## Abstract

**Background & Aims:**

Barrett’s epithelium measurement using widely accepted Prague C&M classification is highly operator dependent. We propose a novel methodology for measuring this risk score automatically. The method also enables quantification of the area of Barrett’s epithelium (BEA) and islands, which was not possible before. Furthermore, it allows 3-dimensional (3D) reconstruction of the esophageal surface, enabling interactive 3D visualization. We aimed to assess the accuracy of the proposed artificial intelligence system on both phantom and endoscopic patient data.

**Methods:**

Using advanced deep learning, a depth estimator network is used to predict endoscope camera distance from the gastric folds. By segmenting BEA and gastroesophageal junction and projecting them to the estimated mm distances, we measure C&M scores including the BEA. The derived endoscopy artificial intelligence system was tested on a purpose-built 3D printed esophagus phantom with varying BEAs and on 194 high-definition videos from 131 patients with C&M values scored by expert endoscopists.

**Results:**

Endoscopic phantom video data demonstrated a 97.2% accuracy with a marginal ± 0.9 mm average deviation for C&M and island measurements, while for BEA we achieved 98.4% accuracy with only ±0.4 cm^2^ average deviation compared with ground-truth. On patient data, the C&M measurements provided by our system concurred with expert scores with marginal overall relative error (mean difference) of 8% (3.6 mm) and 7% (2.8 mm) for C and M scores, respectively.

**Conclusions:**

The proposed methodology automatically extracts Prague C&M scores with high accuracy. Quantification and 3D reconstruction of the entire Barrett’s area provides new opportunities for risk stratification and assessment of therapy response.

## Graphical abstract

**Figure:**
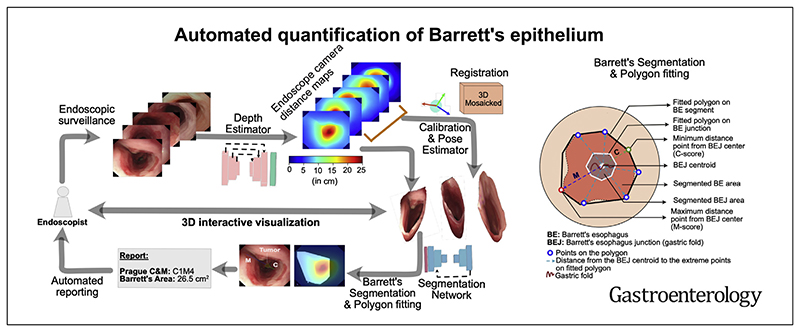

Barrett’s esophagus (BE) is a precancerous condition associated with an annual progression rate to esophageal adenocarcinoma (EAC) of 0.12%–0.13% per year.^1,2^ The widely established Prague classification indicates the circumferential length (C) and the maximal length (M) of the extension of Barrett’s epithelium from the top of the gastric folds into the distal esophagus (Figure 1). The Prague classification is recommended as a risk stratification tool to determine the interval for surveillance endoscopy^3^ in US, European, and British guidelines.^4–7^ However, the adherence to the guidance varies from 22% to 69%^7–10^; it is followed more often in academic than in community settings^8,9^ and by endoscopic interventionalists than by diagnostic endoscopists.^10^ The Prague score is minimally quantitative, subjective, and subject to operator dependence, with difficulties in determining the “top of the gastric folds” owing to differences in insufflation.

The annual progression rate to adenocarcinoma is significantly higher for patients with BE segment ≥3 cm (0.25% per year) than for short BE length <3 cm (0.07% per year).^11^ Therefore, guidelines recommend surveillance intervals based on BE length.^4,6^ Islands of columnar lined epithelium are ignored in the Prague classification but are encountered in one-third of patients with BE; in about half of those, the islands are located proximal to the farthest extent of the Barrett’s segment and can be large, especially after radiofrequency ablation.^12^ Barrett’s islands can harbor dysplasia or EAC, and their histology upgrades the overall Barrett’s epithelium dysplasia grade in 15.7% of cases.^12^

Because current endoscopic surveillance programs are costly, time consuming, and poorly adhered to, better risk stratification of patients with BE to tailor surveillance recommendations is highly desirable. To date, automated, quantitative assessment of the Barrett’s length and area for risk stratification, or for monitoring the response to ablative therapy by comparing pre- and post-treatment extension is not available. Furthermore, our quantitative understanding of the temporal evolution of BE and response to treatment is still limited. A research and clinical tool that provides automatic quantitative assessment of the Barrett’s area and allows spatiotemporal monitoring of topographic changes would be extremely helpful.

The present study aimed to evaluate the accuracy of assessing the Prague classification and the Barrett’s area quantification automatically by generating 3-dimensional (3D) reconstruction of the esophageal surface from 2-dimensional (2D) endoscopic video images by leveraging camera-distances from the gastric folds. Effectively, this 3D reconstruction provides an extended field of view which can also be used for clinical reporting and review. Building on advanced computer vision techniques, our algorithm was trained on simulated as well as on real patient data.

## Material and Methods

### Setting and Design

This study was performed at the Translational Gastroenterology Unit at the Oxford University Hospitals NHS Foundation Trust, a tertiary referral center for endoscopic therapy of BE neoplasia, and at the Horton General Hospital in Banbury, UK. Patients with known BE coming for endoscopic surveillance or endoscopic treatment were included in this study. The study was approved by the local Research Ethics Committee (Ref. 16/YH/0247).

High-definition videos from white-light endoscopy and narrow-band imaging were prospectively recorded with the use of Olympus endoscopes (GIF-H260, EVIS Lucera CV260, and GIF-H290; Olympus Medical Systems, Tokyo, Japan). HUB IMH-20 (Olympus Medical Systems) HD video recorders were used to record videos in the MP4 format. Measuring and subtracting the distances from the tip of the inserted endoscope at the top of the gastric folds and at the proximal squamocolumnar margin to the incisors was used to endoscopically assess the circumferential and maximal length of the BE measurements. Prague C&M scores were reported for all endoscopies in patients with BE.

### Datasets

#### Endoscopy patient cohort

**Figure 2:**
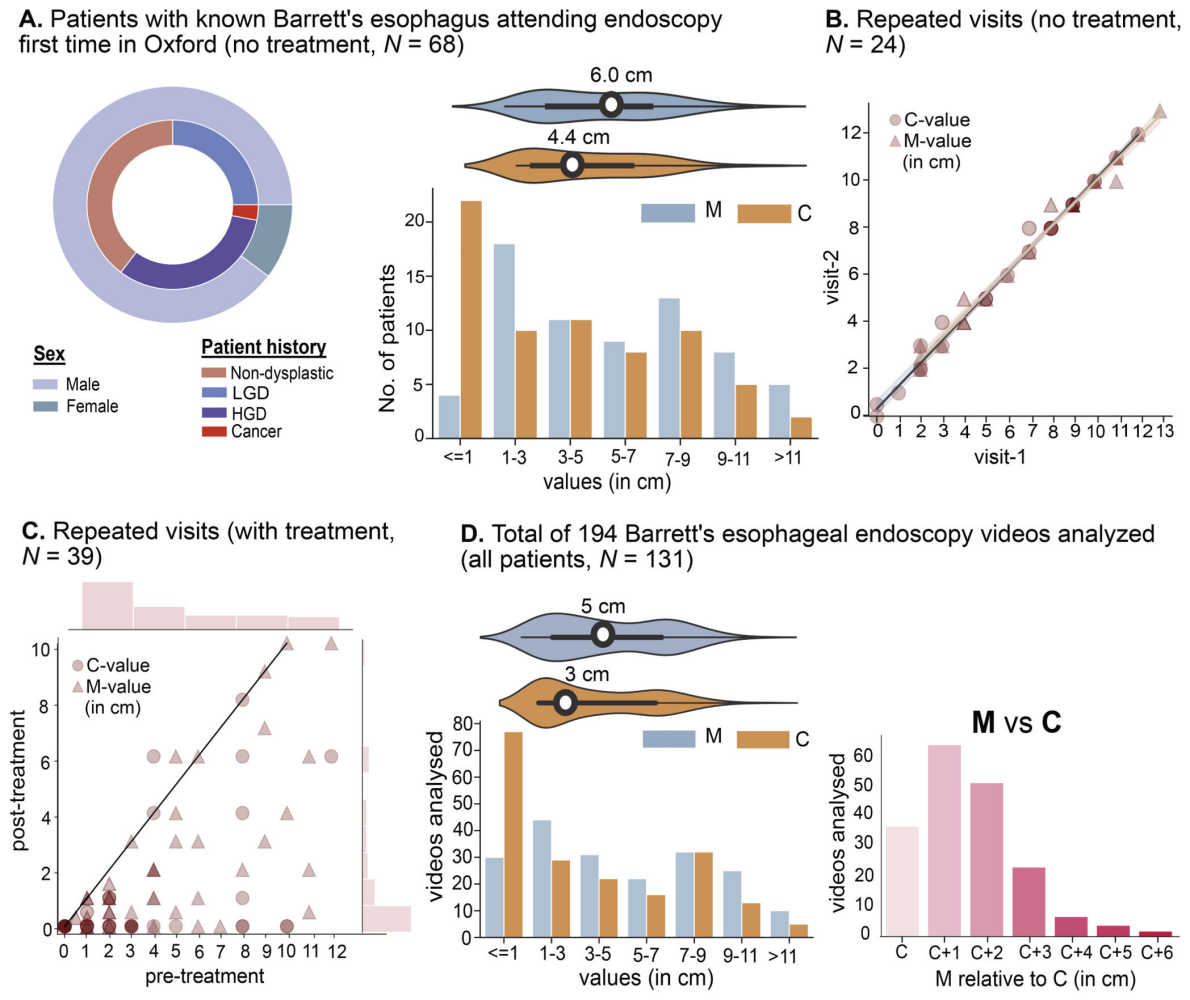
Patient cohort used for clinical evaluation study. (**A**) 68 patients attending for their first visit (61 male and 7 female) with different histology (left, second ring of pie-plot). Prague C and M score variation in Barrett’s length (right top, violin plot) and number of patients for short (≤1, 1–3), medium (3–5, 5–7, 7–9), and long (9—11, >11) Barrett’s esophagus C and M values (right bottom). (**B**) C and M scores measured at 2 consecutive visits after an average of 6 months (without treatment; n = 24). (**C**) Prague C and M scores before and after treatment (n = 39). Most post-treatment scores show reduction in Barrett’s extension. (**D**) On the left, C & M values recorded for all videos analyzed in this study with variations as violin plots (top). On the right, lengths of maximum-length M are provided relative to C with subsequent increment of 1 cm on C for each next label.

The endoscopy patient cohort investigated was split into 3 different groups (Figure 2): dataset 1: 68 newly diagnosed patients with presence of BE attending their first endoscopy before treatment; dataset 2: 24 patients with BE not having received endoscopic treatment between two consecutive endoscopy visits; and dataset 3: 39 patients with BE receiving endoscopic treatment for comparison of pre- and post-treatment measurements.

The Prague score measurements were endoscopically determined by 2 expert upper gastrointestinal (GI) endoscopists. The majority of the patients in this cohort were male (89.7%); the average age of all patients was 67.5 years. For the first visit, variable sizes of M and C scores can be observed with a mean size of 6 cm for the M-value and 4.4 cm for the C-value (Figure 2A). The majority of patients (22 patients) had a C-value <3 cm. Reporting of the Prague C&M values was consistent in repeated visits with a marginal deviation only (Figure 2B). The pre- and post-treatment dataset provided the evidence that the majority of the patients had significantly reduced Prague C&M values in the post-treatment measurement (Figure 2C). A total of 194 videos from 131 patients were analyzed (Figure 2D). The patient cohort included variable maximum length “M” relative to circumferential length *“C”* (Figure 2D, right). Five patient videos from dataset 3 were also used for measurement of BEA.

#### Simulated endoscopy data for training

A 3D phantom model of 18.75 cm length and 2 cm internal diameter was first digitally modeled with the information derived from the CT images of a reconstructed esophagus and esophageal endoscopy videos and then printed. To simulate a real-world endoscopy, a point source light next to the camera was modeled in our digital 3D phantom using “Blender” animation software. Video images for both the esophageal surface and corresponding criterion-standard depth maps were computed at 48 frames per second. To tackle illumination variability, both dim (low and medium intensity) and bright diffuse light settings with quadratic attenuation were used to mimic an endoscopy light source inside a hollow organ (ie, varying intrinsic brightness). Several virtually generated endoscopy camera trajectory paths including straight, spiral, zigzag with small rotations (1 to 45 degrees), and inclinations (1 to 30 degrees) in both forward and reverse directions were used (Supplementary Figure 2A). These paths also mimic the interactions of light with tissue in the absence of natural light.^13^ The acquired images were 3-channel (RGB), and depth maps were 1-channel data of size 256 × 256 pixels. In the depth image, each pixel position corresponded to the distance of the endoscopic camera to the esophageal surface in mm.

To test the quantification and 3D reconstruction, BE patterns were printed inside of the printed phantom with the use of a dark pink–colored silicon coating, and normal squamous area was represented by a light pink color (Supplementary Figure 2B). Ground-truth measurements for Prague C&M and island lengths were acquired with the use of Vernier calipers and for BEAs with mm grid paper. Endoscopic phantom videos were acquired with the same gastroscope used for patient examinations.

### Artificial Intelligence for Computer-Aided Barrett’s Quantification System

The primary goal is to assist endoscopists in acquiring robust and reliable Prague C&M scores automatically. The system also computes BEA, which can be a helpful indicator for measuring risk in patients with large island segments. By mapping 2D video images to a 3D reconstruction, we present a novel approach of performing a comprehensive risk analysis of Barrett’s patients that was previously not possible.

**Figure 3:**
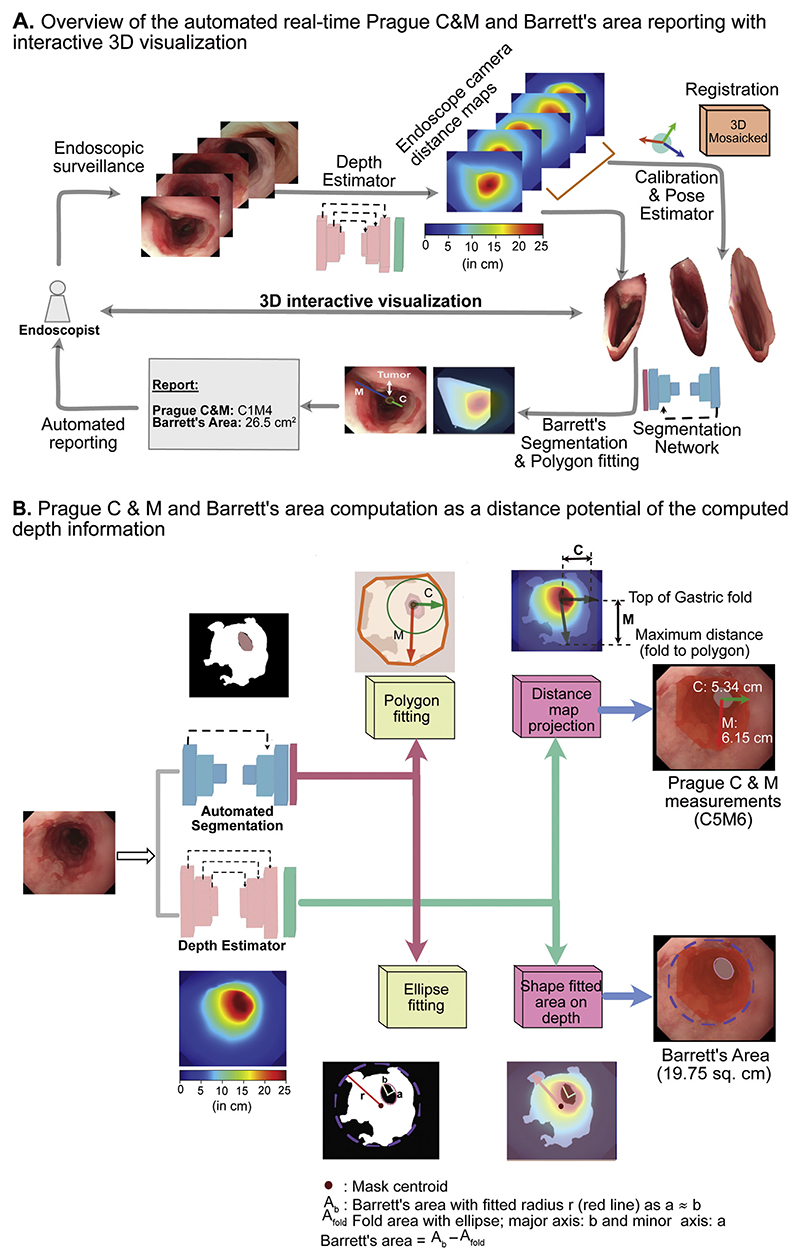
Barrett’s quantification system. (**A**) Block diagram representing the acquisition and computational flow for both reconstructed 3D views and automated Prague C&M and Barrett’s epithelium area (BEA) reporting system. (**B**) Real-time computation of Prague C&M criteria and BEA measurements directly from the depth estimation (online). Top: C and M measurements; bottom: BEA measurement. Both share common depth estimator and segmentation models. Simple ellipse fitting is used to estimate real-world measurements (cm for Prague C&M and cm^2^ for BEA).

Figure 3A illustrates an overview of the artificial intelligence (AI) system design of our proposed Barrett’s quantification system. The system measures the Prague C&M scores, leveraging the learnt endoscopy camera distance estimation. A deep learning–based depth estimator network (Supplementary Figure 1) was used to generate a distance-from-camera, ie, depth map. These depth maps enable projection of 2D endoscopy images onto a 3D space, allowing conversion from pixel to real-world mm measurements. For paired endoscopy images, camera position and orientations were computed based on their estimated depths and initially acquired camera focal length and camera center. This enabled alignment of the two 3D projected images as a mosaicked 3D surface. However, this was needed for only a few cases (8/194 videos with C&M values >11 cm). Furthermore, deep learning–based segmentation (Supplementary Material) was applied together with the shape fitting (Figure 3B and Supplementary Figure 3) to compute both Prague C&M and BEA for the clinical reporting.

Figure 3B details each step for Prague C&M and BEA measurements. All masked pixels (BEA in our case) are fitted with a convex polygon (called the convex hull). Polygons are fitted to the segmented BEA and gastric junction areas. Euclidean distance is measured between the center of the gastric junction and the extrema of the polygons (detailed in Supplementary Figure 3). The smallest distance is considered as the radius of the circumferential measurement C, while the maximal Barrett’s length M is based on furthest distance point. Depth maps that provide the distance estimates are then used to compute the Prague C&M in cm (Figure 3B, top), ie, distance on the computed depth map for length between the center of the gastric junction to the located distal points on the fitted polygon). Similarly, for area measurements we estimate ellipse axes outside of the fitted polygon such that each pixel is taken into account (Figure 3B, bottom). Areas for the BEA (A_b_) and the gastric fold area (A_fold_) are computed and the depth maps are used to estimate their 3D measurements (in cm^2^) as distance projections. A difference value (A_b_ — A_fold_) for A_fold_ >1 cm^2^ provides the final estimate of the BEA. These computations depended on the following assumptions: 1) the esophagus must be sufficiently insufflated for a fraction of a second and the gastric folds are visible, and 2) the endoscopic camera is held nearly perpendicular to the gastric junction.

In the case that these assumptions are violated, eg, a large (>11 cm) Barrett’s segment with invisible gastric folds, we adhere to the quantification performed by our system in Figure 3A. In all cases, the computed depth maps also allow for an efficient 3D reconstruction.

### Testing Criteria

We assessed the accuracy of the designed system by computing the difference, relative error, and root mean square error (RMSE) metrics between automated and ground truth measurements (both on phantom and patient data).

Relative error, and RMSE metrics (Supplementary Material) are used to quantify the maximal diameter of an island, Prague C&M, and BEA measurements on the phantom endoscopy data compared with the ground-truth measurements of the painted BE in the phantom.

For the testing on the patient data, the mean difference and relative error for each short, medium, and large C&M categories are reported. These quantify the measurement variability between our automated system compared with the expert endoscopist measurements.

Standard deviation for repeated measurements on phantom data is reported for precision.

### Statistical Analysis

In the absence of reference data from previous studies on Barrett’s quantification, no formal sample size calculation was carried out. Endoscopic Prague C&M quantification was carried out independently from the simulated data measurements. For this we grouped first visit patients according to the reported C and M lengths. To increase the statistical power of the analysis we computed Cohen’s *k* and Spearman correlation *r_s_* for the entire group and separately for C and M scores. These statistical measures provide inter-rater reliability. Higher values (0.81–1.00) suggest almost perfect agreement between the two measurements (expert and automated), and values in the range of 0.41–0.60 indicate moderate agreement. Paired *t* tests were performed to compute the significance of any differences between our automated Prague C&M measurements and the expert-acquired values, for each Prague category.

## Results

### Training of the Automated System

We used a feature pyramid–based depth estimator that uses multi-scale feature pooling (Supplementary Figure 1), enabling it to preserve information critical for predicting camera distances. Ten thousand simulated images acquired from a digital phantom were used for training. Only the digital phantom model was used for training without any texture transfer. An artificial pink coating (Supplementary Figure 2B) was used to mimic Barrett’s area. Diffuse light incidence (devoid of natural illumination in an enclosed organ such as the esophagus) was exploited for different trajectory settings. Twenty percent (2000 synthetic image and depth pairs) of the training data was used for validation and refinement during training which helped the network to obtain optimal hyper-parameter and avoid overfitting.^14^ All training was done for 1000 epochs (~100 hours) on an NVIDIA GeForce RTX 2080Ti with a batch size of 8. The average inference time on a 256 × 256 image was 0.03 seconds. Further implementation details of segmentation is provided in the Supplementary Material.

### Testing on 3D Printed Phantom Endoscopy Data

The results for the automatic quantification of C&M values, maximal island diameter, and BEA from the esophageal endoscopy videos done on the 3D printed phantom are presented in Table 1. In 5 different video frames of the phantom, the proposed method achieved an average accuracy of 97.2% (2.8% relative error) and an average deviation of only 0.9 mm from the ground-truth measurements. In addition, the RMSE was estimated to be 1.2 mm, confirming a substantial agreement (k of 0.72 and *r_s_* of 0.99) with the ground-truth measurements. Small standard deviations in 5 repeated automated measurements suggest greater precision compared with manual measurements. Table 1 also demonstrates the validation of the BEA quantification. It can be observed that the standard deviation between measurements was ~1 cm^2^ for BEA 1 and subsequently increased for larger areas. However, the relative error for all automated BEA and island measurements was still <10%. The average RMSE was only 0.39 cm^2^ with *r_s_* of 0.94 and *k* of 0.54.

### Testing on Patient Data

The expert measurements of the Barrett’s length during endoscopy only allow integer cm values for the Prague scores while for automated assessments we have used exact computed measurements (up to mm scale). This is to understand why the automated results deviate from the crude endoscopists’ measurements. Small deviations (0–0.5 cm) are thus to be expected. We calculated the mean difference and relative error for each dataset (Table 2) and for the entire patient cohort (Supplementary Table 1).

**Figure 4:**
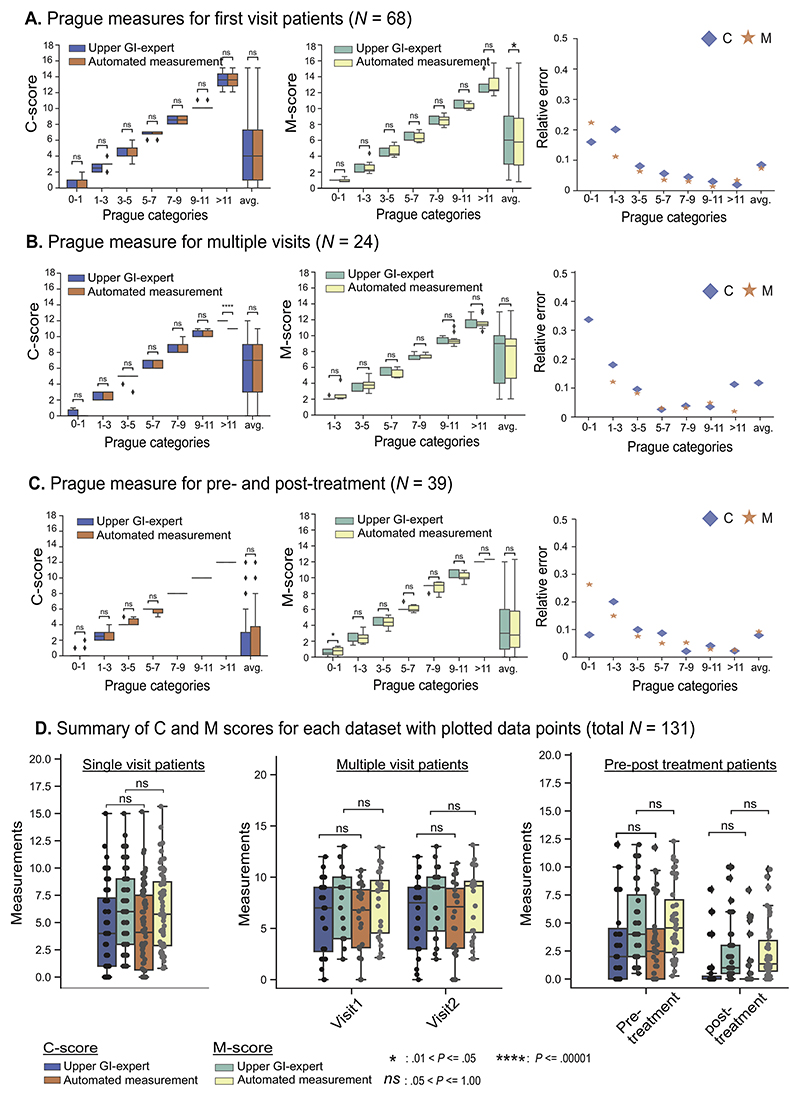
Measurement of Barrett’s C and M scores in 131 patients: comparison between Prague C and M scores reported by senior upper GI endoscopists and the automated measurements from our system. Nonsignificant comparisons (paired *t* test: *P >* 0.05) iares marked as ns. (**A**) Prague C and M measurement comparison for first visit patients (n = 68). (**B**) Expert upper GI endoscopists and automated Prague scores comparison for 2 consecutive patient visits (n = 24) without receiving any treatment. (**C**) Pre-treatment and post-treatment measurements (n = 39) are presented. (**D**) Box plot for each dataset for C and M measurements for both expert and automated measurements.

Figure 4 and Table 2 present the comparison between C and M scores logged by the upper GI expert endoscopists and the automated measurements on patient data. Larger deviation in both C and M scores can be observed for most automated measurements compared with expert logged values for short (0–1 cm, 1–3 cm, and 3–5 cm) C and M segments (Figure 4A-C). For the first-visit patients (dataset 1; Figure 4A), Prague categories for short C and M recorded higher deviations in small and large measurements (larger error bars), resulting in larger relative error compared with other categories (eg, 0.16 and 0.22 relative errors for C and M, respectively, for the 0–1 cm category in Table 2). However, the mean difference for all categories is still <0.5 cm (Table 2). The median lines in the box plots (Figure 4) are close to the expert measurements. Similarly, for multiple-visits patient data (Figure 4B), large relative errors are obtained for C and M scores for short lengths (Table 2). A similar trend is found for the pre-treatment data (relative error of 0.19 in C for 1–3 cm category and 0.32 in M for 0–1 cm category; Table 2) and post-treatment data (relative error of 0.23 in M for 0–1 cm category; Table 2).

In general, mid- and high-range categories (5–7, 7–9, 9–11, and >11) showed the smallest relative errors, except for the >11 cm category for C score in dataset 2 (visit 2), which showed 1.27 cm difference but on single data point (Table 2), and for the 9–11 cm category for dataset 3 (post-treatment), with mean differences of 0.49 in C score and 0.53 in M score. Most recorded relative errors were marginal, approximately <0.15, ie, an error of 15% in measurement. *P* values for paired *t* test conducted for all measurements showed that for almost all categories the obtained results did not deviate significantly, suggesting nonsignificant change between the two measurements, including for short categories (Figure 4D). The statistical agreement measured for dataset #1 was *k* = 0.820 and *r_s_* = 0.992 for C score and *k* = 0.903 and *r_s_* = 0.997 for M score, showing substantial to almost perfect inter-rater reliability. Similarly, for datasets 2 and 3, substantial (0.61–0.80) to almost perfect (0.81–1.00) inter-rater reliability was achieved.

For the entire patient cohort (Supplementary Table 1), the overall relative errors (mean difference) were 8% (3.6 mm) and 7% (2.8 mm) for C and M scores, respectively. Even though the majority of patients reported 0–1 cm in C and 1–3 cm in M (77 and 44 patients, respectively), the relative error was small (0.12). The highest relative errors were observed in the 0–1 cm sub-group for M (0.24) and in the 1–3 cm sub-group of C (0.19). Although the mean differences for most categories were again <0.5 cm, in the >11 cm sub-group for C it was 0.69 cm, but averaged over 5 patients. Almost perfect inter-rater reliability was achieved (*k* = 0.84 and *r_s_* = 0.99 for C and *k* = 0.87 and *r_s_* = 0.99 for M).

Supplementary Table 2 demonstrates the applicability of the area measurement to quantifying the efficacy of the ablation therapy in 5 patients with BE. It can be observed that even though the C and M measurements are reduced for all 5 patients, in most cases the residual Barrett’s areas/islands still measure >10 cm^2^ after the first ablation, and in 1 case it is as large as >26 cm^2^, although reported C and M are zero.

### Qualitative Assessment of the System Outputs

**Figure 5:**
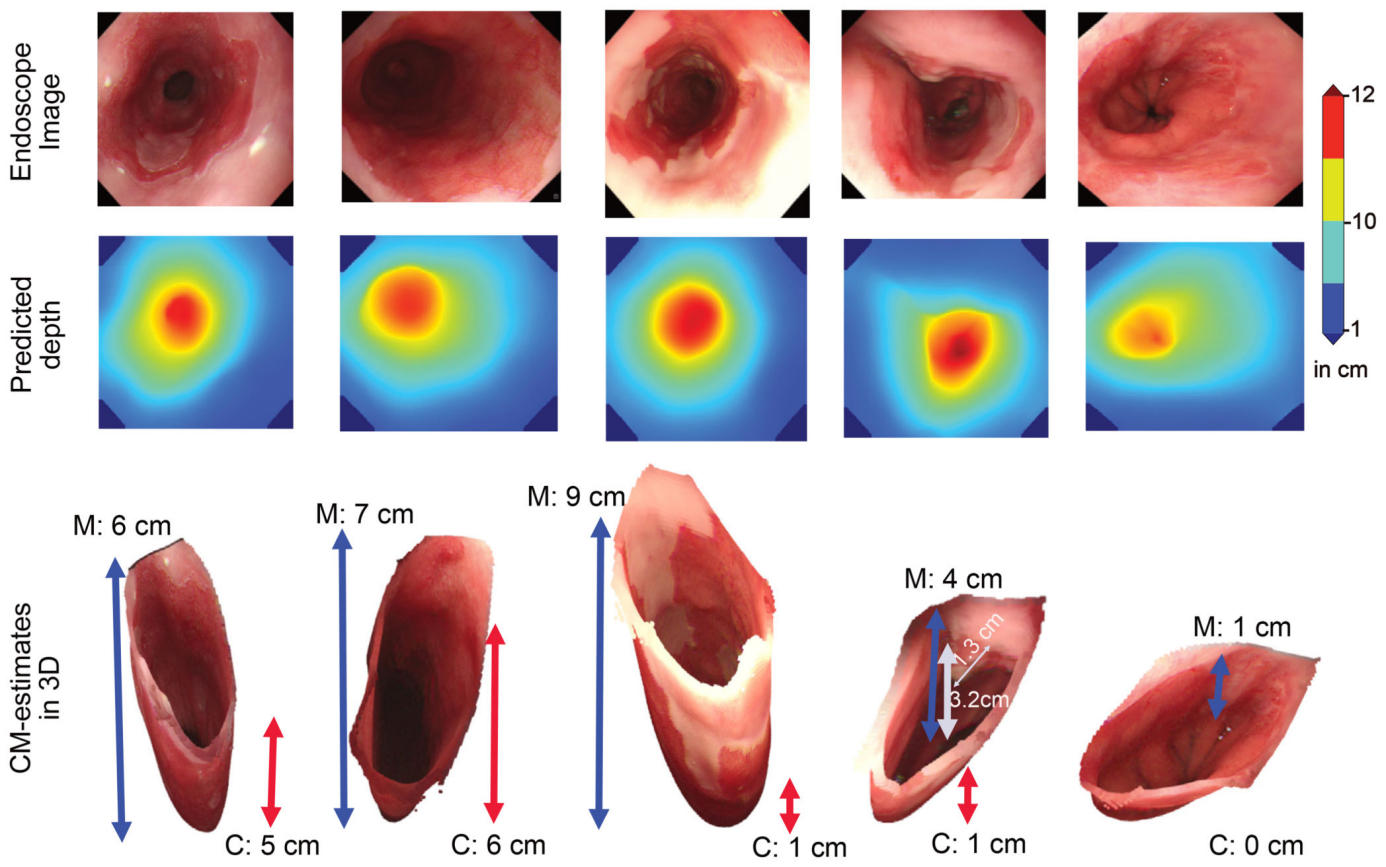
Depth-from-camera estimation and Prague C&M measures: Predicted depth maps (middle) for real gastroesophageal endoscopy frames in 5 patients (top). The Prague C&M values in 3D-reconstructed esophagus are also shown (bottom, scaled for visualization). The 3D esophagus reconstruction enables the measurement of encountered lesions and relative distances of biopsy spots in relation to the top of the gastric folds (eg, the small nodule in the image in the 4th column is 1.3 cm in size and ~3.2 cm from the gastric folds).

Figure 5 illustrates the predicted depth maps and the corresponding 3D views with their respective C and M values for 5 unique patient endoscopy frames. The Barrett’s area segmentation, polygon fitting, and mapping of predicted depths (Figure 3B) have been applied for real-time estimation of the Prague C&M. Supplementary Figure 4 represents the automated measurement of BEA for pre- and post-treatment in 2 unique patients. It can be observed that the large island after treatment in the first patient recorded an area of 26.73 cm^2^.

## Discussion

The proposed methodology evidently provides a quantitative, reliable, and automated assessment of Barrett’s length and area suitable for routine clinical use. Although Prague classification has been shown to be reproducible,^15^ it is fundamentally limited as an accurate measure of disease extent through subjective estimation of 2D measurements of a Barrett’s segment that is frequently nonuniform in shape. Excluding Barrett’s islands, the Prague classification provides only a surrogate measure and not a true quantitative analysis of the total area of BE. In the present study, we developed and independently tested a real-time AI system that automatically identifies, delineates, and quantifies BE with high reproducibility by recognizing the typical landmarks at the top of the gastric folds and the proximal squamocolumnar margin.

We present a systematic analysis of the automated measurement performance by comparing it with reference data acquired by endoscopy in a purpose-built phantom as well as patient endoscopic video data with Prague scores provided by expert endoscopists. Reliability of the proposed depth measurement algorithm against both phantom and patient data suggests accurate and precise measurement capability of the designed AI model. The Prague C&M scores reported by the upper GI specialists correlated well with the automatically measured values on patient data (*r_s_* of 0.886–0.997) and a substantial statistical agreement (k of 0.61–0.80) up to almost perfect (*k* 0.81–1.0) for each dataset (Table 2 and Supplementary Table 1). In general, mean differences for most estimated sub-categories, including short, medium and long C&M, were <5.3 mm. Even though relative errors in short Prague categories (eg, 0–1 cm and 1–3 cm) were observed to be higher, they were <0.32 mm in most cases.

Furthermore, the quantitative validation on the endoscopic phantom video data demonstrated >97.23% (2.77% relative error) accuracy with only ±0.9 mm average deviation, *k* = 0.72 and *r_s_* = 0.99, from the available groundtruth measurements (Table 1); this implies that the computer-aided measurement of the Prague scores, which provides measurement in mm range, is more precise than the measurement by the expert upper GI endoscopists during endoscopy.

Repeated measurements of Prague C&M over 2 visits showing no change in Barrett’s length was consistent with the expert finding (Figure 4D, center). Similarly, for patients undergoing 1 session of radiofrequency ablation (Figure 4D, right), a reduction in the length of the Barrett’s segments (mostly C) at the next visit was recorded by the system similarly to the endoscopists. Both of these results provide evidence of system repeatability and reproducibility, and the latter also demonstrates its strength for accurate quantification of therapy response. Paired *t* test on each of these measurements showed no statistically significant difference between the expert and automated measurements.

Existing clinical studies indicate that an accurate and more systematic assessment of the Barrett’s area would be of clinical value. Anaparthy et al^16^ demonstrated that with every centimeter increase in M score of Barrett’s, the risk of progression to high-grade dysplasia or EAC increases by 28% (*P* = 0.01). Barrett’s segment ≥3 cm showed significantly greater prevalence of dysplasia (23% vs 9%; *P* = 0.0001).^17^ A recent meta-analysis demonstrated that non-dysplastic short-segment BE has significantly lower rates of neoplastic progression than long segments.^18,19^ It is thus critical to report precise measurements. In addition, measurement of the BEA should be incorporated in reporting to measure risks more reliably. The built technology allows for mm-scale measurements of both Barrett’s lengths and areas. Sharma et al^20^ demonstrated that recurrence of intestinal metaplasia after ablation therapy in the form of islands is common, with rates of 8%–10% per patient-year reported by some studies.^21,22^ To address this issue, 3D reconstruction of Barrett’s can be a step forward for effective follow-up of the mucosa.

By applying our system to the quantitative analysis of a patient’s response to therapy we demonstrate that measuring of the entire BE provides further evidence of tangible improvements over the commonly used Prague scores. A patient who received radiofrequency ablation with reported Prague C0M0 score had a considerably large untreated BEA (Supplementary Figure 4). Such insular areas could potentially harbor cancer or dysplasia.^12^ It is therefore eminently important to build technologies that can provide BEA for quantification of therapy response. The validation and reliability tests on 3 phantom endoscopy video data (with known measurements) showed the efficacy of our proposed BEA measurement, with only 0.30 cm^2^ average deviation observed compared with the groundtruth BEA, with moderate agreement (*k* = 0.54) and strong correlation (*r_s_* = 0.99). The study included 2 different island sizes and 3 differently shaped BE areas.

In addition to enabling accurate, precise and systematic measurements of the BEA, the proposed 3D surface reconstruction is likely to revolutionize our way of reporting Barrett’s surveillance endoscopy and corresponding histology requests. By documenting biopsy locations and encountered pathology (Figure 5, 4th column), it provides a visual linkage between endoscopy and any further histopathologic assessment. This compact representation can form the basis for an additional specialist review. The quantification of the entire area of BE is plausibly a better tool for risk stratification to measure progression to Barrett’s neoplasia than the currently used extension in length. Up to now, there is no research tool available to investigate and quantify the emergence of BE over time.

This novel AI system will enable the monitoring of temporal morphologic changes of BE during development or possible regression in response to treatment. Quantification of the BEA can be used to assess treatment efficacy after ablative treatment of dysplastic BE, such as radiofrequency ablation, cryoablation, argon plasma coagulation, or stepwise endoscopic resection.

Other groups have introduced AI systems for computer-assisted recognition of early esophagus cancer and dysplasia, mainly from endoscopy still images, but recently also for real-time endoscopy.^23–26^ Our proposed system can automatically detect, delineate, and quantify BE during endoscopy and generate a 3D reconstruction of the individual esophageal anatomy. The 3D reconstruction allows for a visual interaction with any area of interest, projecting it back to the spatial distribution in the anatomical context. We envisage building a clinical decision support tool combining pattern-recognition systems with our quantification and reconstruction tool to enable a more comprehensive investigation of the esophageal mucosa surface.

This was a single-center pilot study of a new technology and therefore requires further evaluation in the clinical and nonexpert endoscopy settings. Furthermore, our experiments show that BEA estimation can be affected by imaging conditions, organ deformation, and operator variability (Supplementary Table 3) so a careful selection of endoscopic frames is required. Measurement of small islands up to <1 mm is also possible, although tiny point size appearances may require manual zooming and input of points for automated measurement (Supplementary Table 4). To date, we have not evaluated the automatic quantification of Barrett’s extension and reconstruction from endoscopy against measurement after surgical esophagectomy. However, the surgical resection specimen will also be subjected to shrinking artifacts and contractions. Although the study included only Olympus endoscopes, the use of digitally acquired synthetic data for training without the need for transfer learning of texture should enable the same efficacy with endoscopes from other manufacturers.

In conclusion, we present a deep learning–based AI system that reliably quantifies the extent of BE in real time. It holds the potential of enhancing endoscopy reporting by providing quantitative and objective data that can be used for review and the assessment of disease progression.

## Supplementary Material

Supplementary Material

## Figures and Tables

**Figure 1:**
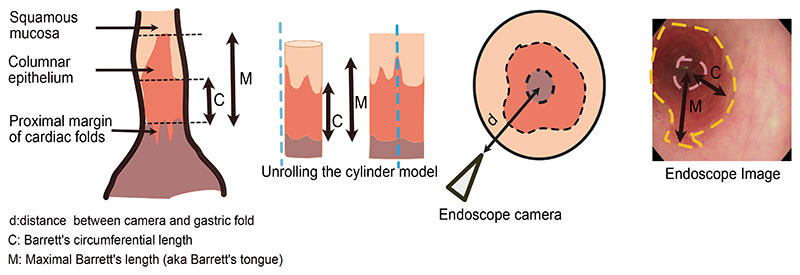
Prague classification. Esophageal squamous and columnar cell linings depicting Prague C&M measurements taken from top of gastric folds up to the squamocolumnar junction in 3D (left) and 2D endoscopic (right) images.

**Table 1:** Automated Barrett’s Length and Area Quantification Using Endoscopy Data Acquired From the 3D Printed Phantom

Variable	Barrett’s markers	Measurements			Average Errors			Agreement
		Ground Truth, Mean ± SD	Expert Endoscopist	Automated Measurement, Mean ± SD	Abs. Difference	Rel. Error, %	RMSE	
Length measure, cm	Ma	7.00 ± 0.20	7.00	6.97 ± 0.09	0.03	0.40	0.12	*r_s_* = 0.99
	Mb	6.57 ± 0.21	7.00	6.32 ± 0.29	0.25	3.83		*k* = 0.72
	C	2.34 ± 0.15	3.00	2.31 ± 0.09	0.03	1.28		
	Island 1	2.03 ± 0.11	2.00	2.10 ± 0.05	0.07	3.45		
	Island 2	1.43 ± 0.23	1.00	1.36 ± 0.19	0.07	4.89		
	Overall (average)	3.88 ± 0.19		3.81 ± 0.14	0.09	2.77		
Area measure, cm^2^	Barrett’s Area 1	62.19 ± 1.26	NA	62.62 ± 1.06	0.43	0.78	0.39	*r_s_* = 0.99
	Barrett’s Area 2	69.05 ± 0.84	NA	69.23 ± 2.90	0.18	0.06		*k* = 0.54
	Barrett’s Area 3	76.25 ± 0.73	NA	76.98 ± 3.04	0.73	0.95		
	Island 1	2.30 ± 0.20	NA	2.25 ± 0.16	0.05	2		
	Island 2	2.90 ± 0.26	NA	3.01 ± 0.48	0.11	3.93		
	Overall (average)	36.10 ± 0.56		35.72 ± 0.20	0.30	1.57		

Lengths and areas of differently shaped painted Barrett’s epithelium in the phantom were measured with the use of Vernier calipers and mm-scale grid paper (ground truth; Supplementary Figure 2B). Mean and standard deviation for 5 measurements are provided.

Abs, absolute; k, Cohen’s kappa; NA, not available; *r_s_*, Spearman correlation; Rel, relative; RMSE, root mean square error.

**Table 2:** Mean Difference and Relative Error: Automatic Measurement and Endoscopic Prague Scores Logged by Experts for the Patient Data for 3 Dataset Subcategories Based on the Barrett’s Length

Dataset	No. of Patients		Prague Cat.	Average Expert Score, cm		Average Automated, cm		Mean Difference, cm		Average Rel. Error	
	C	M		C	M	C	M	C	M	C	M
Dataset 1 (n = 68)	22	4	0-1	0.36	1	0.31	1	0.16	0.22	0.16	**0.22**
	10	18	1-3	2.50	2.39	2.79	2.55	**0.42**	0.27	**0.20**	0.11
	11	11	3-5	4.54	4.45	4.68	4.66	0.37	0.30	0.08	0.06
	8	9	5-7	6.75	6.44	6.74	6.49	0.38	0.24	0.06	0.04
	10	13	7-9	8.50	8.54	8.37	8.43	0.39	0.27	0.05	0.03
	5	8	9-11	10.2	10.37	10.04	10.20	0.32	0.16	0.03	0.02
	2	5	>11	13.5	12.70	13.40	13	0.26	**0.46**	0.03	0.04
C score: *k* 0.82, *r* 0.99; M score: *k* 0.90, *r* 0.99											
Dataset 2, visit 1 (n = 24)	3	0	0-1	0.25	NA	0.0	NA	0.33	NA	**0.33**	NA
	5	4	1-3	2.4	2.25	2.74	2.54	0.34	0.29	0.17	**0.13**
	2	4	3-5	5.0	4.25	5.26	4.48	0.26	**0.46**	0.05	0.11
	3	2	5-7	6.67	6.50	6.53	6.43	0.13	0.25	0.02	0.04
	7	7	7-9	8.42	8.85	8.54	8.78	0.24	0.23	0.03	0.03
	3	5	9-11	10.33	10.60	10.10	10.53	0.28	0.43	0.03	0.04
	1	2	>11	12.0	12.50	10.61	12.26	**1.39**	0.24	0.11	0.02
C score: *k* 0.86, *r* 0.99; M score: *k* 0.80, *r* 0.99											
Dataset 2, visit 2 (n = 24)	3	0	0-1	0.37	NA	0	NA	0.33	NA	**0.33**	NA
	5	4	1-3	2.50	2.62	2.92	3.14	0.42	0.28	0.19	**0.10**
	2	4	3-5	4.67	4.50	4.19	4.34	0.48	0.22	0.12	0.05
	3	2	5-7	6.50	6.50	6.37	6.63	0.22	0.13	0.03	0.02
	7	7	7-9	8.37	9.0	8.58	9.21	0.37	0.31	0.04	0.03
	3	5	9-11	10.33	10.40	10.42	10.57	0.40	**0.53**	0.04	0.05
	1	2	>11	12.0	12.50	10.73	12.44	**1.27**	0.20	0.10	0.17
C score: *k* 0.72, *r* 0.99; M score: *k* 0.85, *r* 0.98											
Dataset 3, pre-treatment (n = 39)	16	6	0-1	0.31	0.91	0.30	1.02	0.12	0.29	0.12	**0.32**
	10	8	1-3	2.50	2.25	2.90	2.37	**0.42**	**0.37**	**0.19**	0.17
	4	10	3-5	4.25	4.40	4.20	4.42	0.21	0.32	0.05	0.07
	0	5	5-7	NA	6.20	NA	6.06	NA	0.33	NA	0.05
	6	4	7-9	8	8.75	8	8.85	0.18	0.35	0.02	0.04
	2	5	9-11	10	10.60	9.60	10.40	0.39	0.19	0.03	0.02
	1	1	>11	12	12	12	12.3	0.26	0.30	0.02	0.02
C score: *k* 0.994, *r* 0.990; M score: *k* 0.939, *r* 0.886											
Dataset 3, post-treatment (n = 39)	33	20	0-1	0.10	0.50	0.10	0.62	0.05	0.14	0.05	**0.23**
	0	10	1-3	NA	2.35	NA	2.45	NA	0.27	NA	0.12
	2	2	3-5	4	4	4.80	4.20	0.04	0.20	0.01	0.05
	3	4	5-7	6	6.25	5.50	6.14	**0.49**	0.26	0.08	0.04
	1	1	7-9	8	9	7.90	8.18	0.06	0.09	0	0.09
	0	2	9-11	NA	10	NA	9.46	NA	**0.53**	NA	0.05
	0	0	>11	NA	NA	NA	NA	NA	NA	NA	NA
C score: *k* 0.69, *r* 0.95; M score: *k* 0.65, *r* 0.98											

Highest mean difference and relative error are in bold. NA, not available.

## What you Need to Know

### Background and Context

The risk for neoplastic progression is associated with the length of the Barrett’s esophagus, which is commonly reported with the Prague C&M classification.

### New Findings

The authors developed a novel AI technique to automatically quantify Barrett’s epithelium during endoscopy and performed validation on a 3D printed phantom and endoscopic videos from patients. The system demonstrated accurate measurements for both Barrett’s length and Barrett’s area. It enables 3D reconstruction of the esophagus from 2D images to assist visualization of the entire organ in correct proportions and spatial context.

### Limitations

Multi-center data were not explored in this study. The system design requires sufficient insufflation of esophagus.

### Impact

The developed quantification system has the potential to revolutionize Barrett’s reporting and surveillance.

## Abbreviations used in this paper

AI: artificial intelligence
BE: Barrett’s esophagus
BEA: area of Barrett’s epithelium
EAC: esophageal adenocarcinoma
