# Supplementary material for: A Pilot Study on Automatic Three-Dimensional Quantification of Barrett’s Esophagus for Risk Stratification and Therapy Monitoring
Source: Gastroenterology. Author manuscript; Available in PMC 2024 Dec 9. (PMC7617121; doi:10.1053/j.gastro.2021.05.059)
Supplement: Supplementary Material [file EMS181860-supplement-Supplementary_Material.pdf]

Supplementary Material

Depth Estimator

The depth estimator network is composed of a residual feature pyramid network (RFPN)[1] with ResNeXt-101 backbone pretrained on imageNet. The FPN allows extracting meaningful features at multiple scales for accurate depth estimation. Depth here refers to the distance values of location or position of surface from the camera. All upscaled layers on the right side of the RFPN are subsequently convolved with a sequence of linear and deformable convolution kernels and rectified linear activation functions. The concatenated feature maps obtained after the upsampling block is finally used to predict the depth map[2] of the input image (Supplementary Figure 1).

Barrett's and Gastric Junction Area Segmentation

We used an encoder-decoder framework with ResNet-50 backbone and atrous separable convolutions (referred to as DeepLabv3+)[3] for segmentation of Barrett's area and the gastric fold. Also, to eliminate small island-like objects, a post-processing step was used to exclude them during estimation. Finally, a polygon was fitted based on the extreme locations on the mask.

Network training. The entire network was trained for 200 epochs with 736 images consisting of 45 unique patient video images and validated on 135 images from 23 unique patient videos. All images were resized to 256 $\times$ 256 pixels. A stochastic gradient descent with a learning rate of 0.01 and a momentum of 0.9 was used.

Result on test data. Our network achieved a Dice coefficient of $>$91% for Barrett's area segmentation and 70% for gastric junction segmentation. The inference time reported was 2.8 ms on an NVIDIA GeForce RTX 2080Ti.

Error Metrics

If P_i^{GT} and P_i^{est} represent ground-truth and predicted measurements, respectively, for N number of samples, then:

$$\text{Average relative error} = \frac{1}{N}\sum_{i=1}^{N}\frac{\left(|P_i^{GT} - P_i^{pest}|\right)}{P_i^{GT}}$$

$$\text{Root mean square error} = \frac{1}{N}\sum_{i=1}^{N}\left(P_i^{GT} - P_i^{est}\right)^2$$

Supplementary Video S1. Supplementary Video S2.

References

1. Lin T, Dollár P, Girshick R, He KK, Hariharan BB, Belongie S. Feature pyramid networks for object detection. IEEE Conf Comput Vis Pattern Recognit 2017:2117–2125.
2. Moukari M, Picard S, Simon L, Jurie F. Deep multi-scale architectures for monocular depth estimation. IEEE Conf Image Process 2018:1884–2019.
3. Chen LC, Zhu Y, Papandreou G, Schroff F, Hartwig A. Encoder-decoder with atrous separable convolution for semantic image segmentation. Eur Conf Comput Vis 2018:833–851.

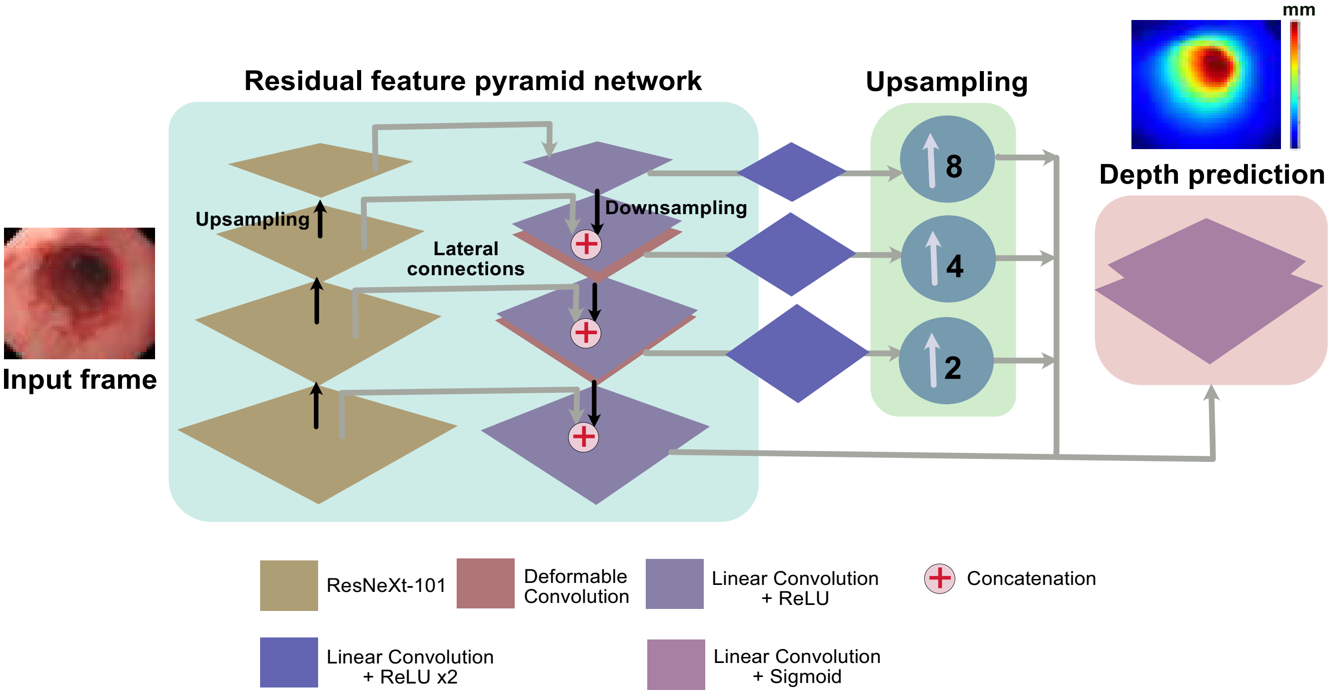

Supplementary Figure 1. Depth estimator network. Proposed deep-learning framework for estimating camera distances (depths) in the endoscopy data. Features are extracted at different layers and learned from to predict the camera distance from each semantically meaningful region, such as the gastric fold and Barrett's junction in our case.

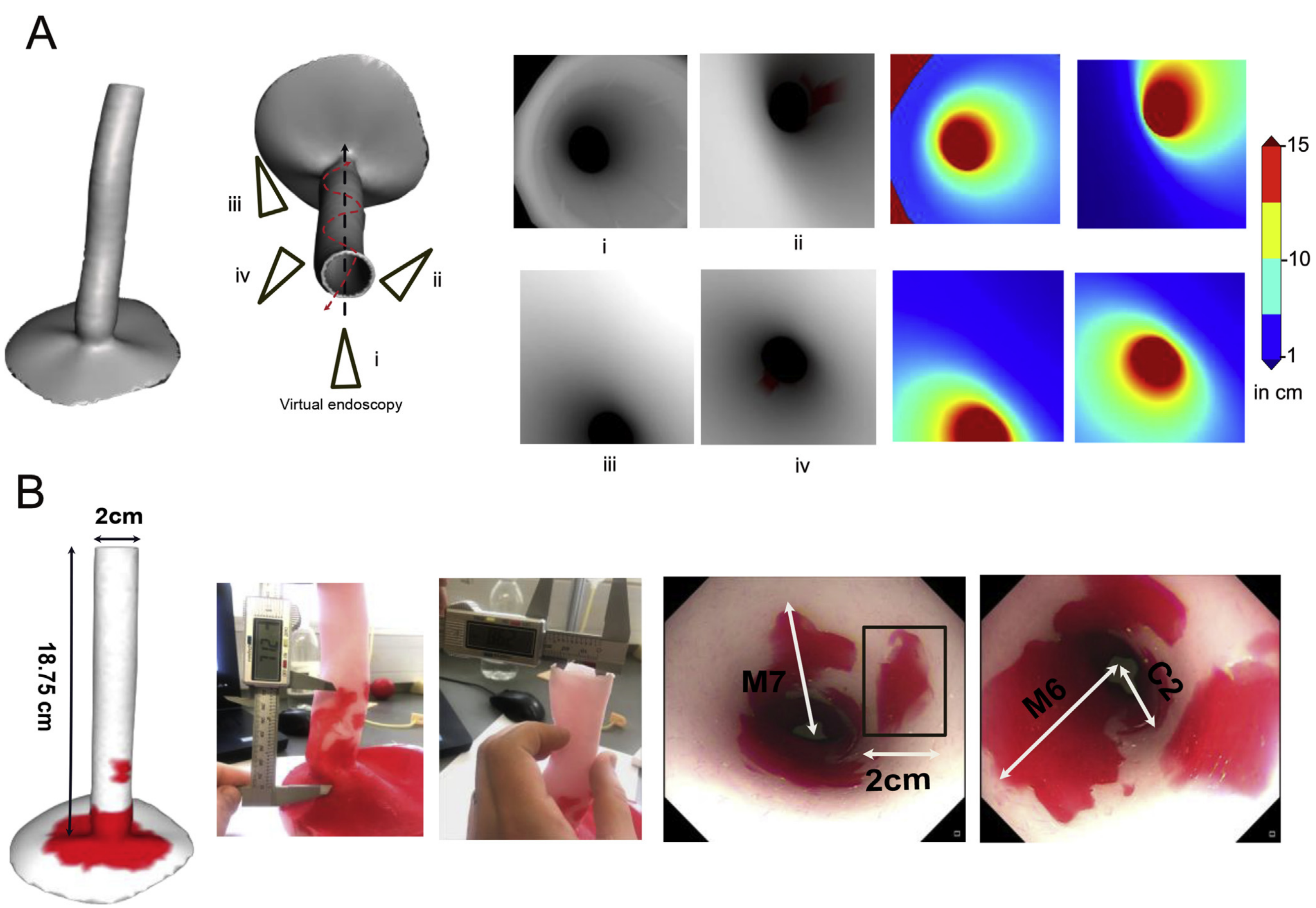

Supplementary Figure 2. (**A**) Simulated depth maps in a virtual 3D esophagus model. Left: Camera trajectories (i–iv) representing straight and spiral camera motion. Right: Endoscopic images and their corresponding depth-map estimation (distance from endoscopy camera). (**B**) Validation data from 3D printed esophagus phantom model with known measurements for C and M (**white arrows**) and island (**black rectangle**). Endoscopy video frames are shown on the right.

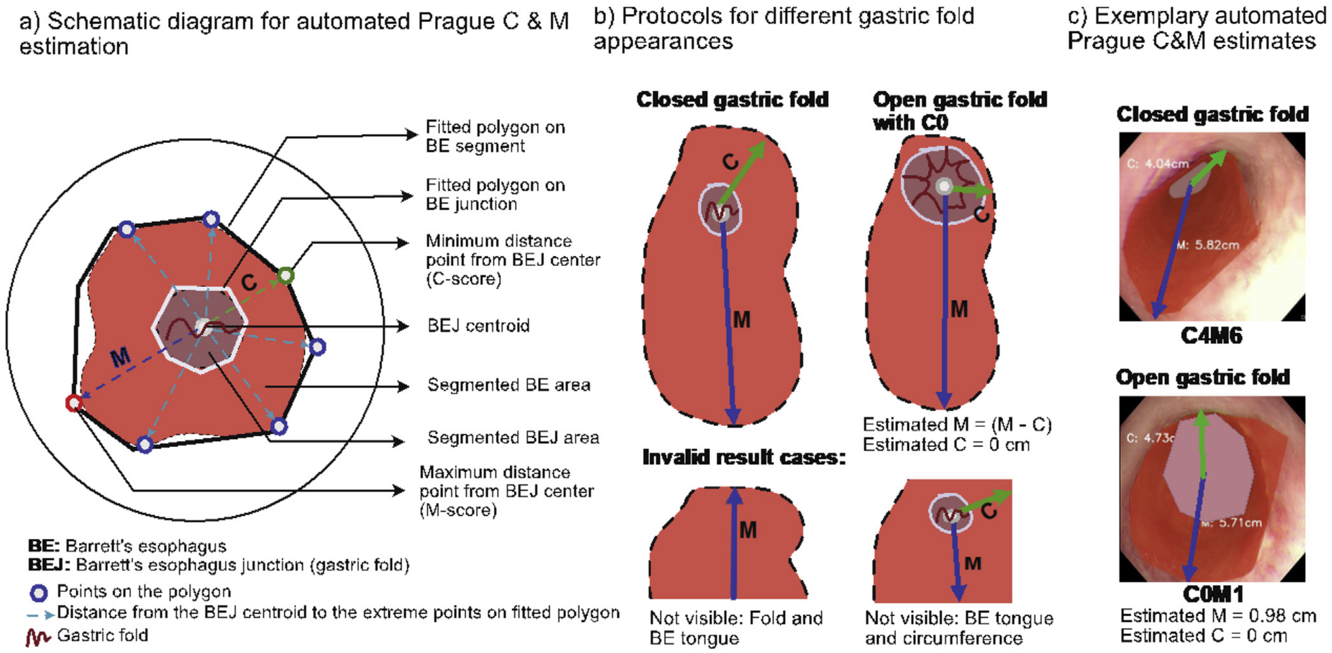

Supplementary Figure 3. Shape fitting and automated measurements for Prague C&M in Barrett's esophagus. (**A**) Schematic diagram representing fitted polygons on the segmented Barrett's area and gastric junction. The circular points at the edges represent the extremum location on this fitted polygon, and the arrows represents the distance from the junction to each point. (**B**) Protocols used to estimate C and M measures at different gastric fold appearances. Top: Closed fold refers to the optimal junction, and open fold refers to the nonoptimal junction, where the final estimate is computed by deducting the junction length. Bottom: Some invalid cases are presented, eg, invisible tongue or fold. (**C**) Illustration of computed Prague C&M from 2 unique patient endoscopy videos for (top) closed gastric fold and (bottom) open gastric fold. For the closed gastric fold case, the distances of both C and M are measured from the center of segmented fold region. But, for open gastric fold with C near to the fold, the computed M is deducted from C for final scoring as in the presented case (bottom), where measure C is 4.73 cm and M is 5.71 cm but owing to the opened gastric junction, the fold is pushed further away, which is deducted from M to obtain C0M1 (C = 0 cm and M = 0.98 cm).

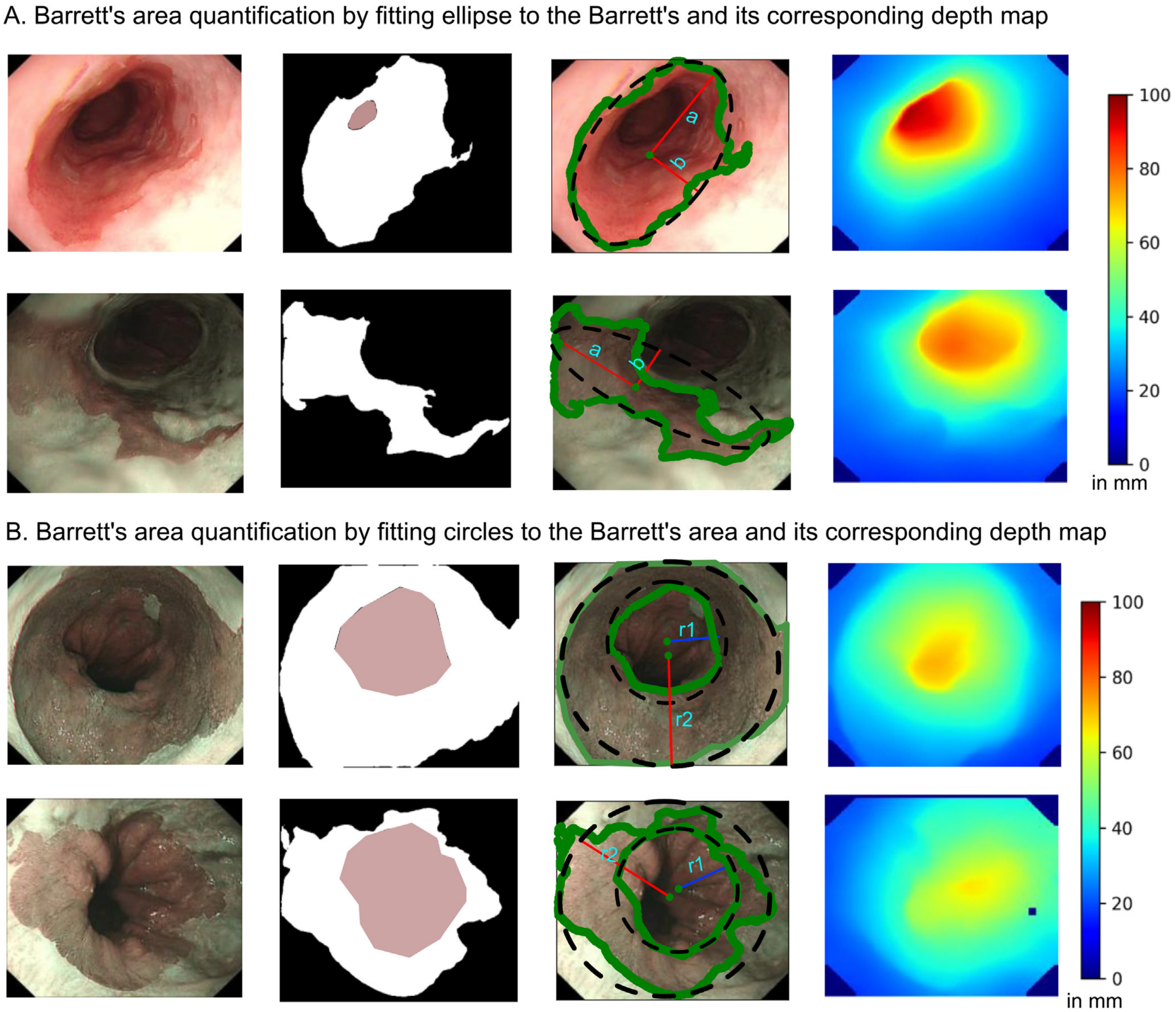

Supplementary Figure 4. Automated measurements for Barrett's area with the use of different parametric shape fittings. (**A**) Elliptical area fitting on the segmented mask of patient 3080 (Supplemental Table 2). Top: Pre-treatment area of 62.05 cm^2; bottom: post-treatment area of 26.73 cm^2. (**B**) Circle fitting on the segmented mask of patient 2006. Two concentric circular area measurements are done to eliminate area around the gastric fold. Top: Pre-treatment area of 47.92 cm^2; bottom: post-treatment area of 5.21 cm^2.

Supplementary Table 1. Mean Difference and Relative Error Provided for All Patients (n = 131)

Dataset	No. of Patients		Prague Cat.	Average Expert Score, cm		Average Automated, cm		Mean Diff., cm		Average Rel. Error	
	C	M		C	M	C	M	C	M	C	M
All Patient Data (n = 131)	77	30	0–1	0.25	0.65	0.20	0.75	0.11	0.18	0.12	**0.24**
	29	44	1–3	2.48	2.36	2.83	2.49	0.41	0.29	**0.19**	0.12
	22	31	3–5	4.50	4.38	4.58	4.49	0.38	0.31	0.08	0.07
	16	22	5–7	6.56	6.36	6.43	6.29	0.33	0.25	0.05	0.04
	32	32	7–9	8.34	8.75	8.38	8.72	0.30	0.30	0.04	0.03
	13	25	9–11	10.23	10.44	10.07	10.33	0.34	0.33	0.03	0.03
	5	10	>11	12.60	12.60	11.97	12.71	**0.69**	**0.35**	0.05	0.03
	131	131			Total average.			0.36	0.28	0.08	0.07
						C score: *k* 0.84, *r* 0.99; M score: *k* 0.87, *r* 0.99					

Statistical methods to test agreement between 2 ratings for each C score and M score (expert endoscopists and automated measurement) are also provided. For achieving high power (>90%) and confidence (>90%), statistical measurement have only been done for the group and not for the Prague categories individually. Highest mean difference and average relative error are in bold.

Supplementary Table 2. Automated Pre- and Post-treatment Barrett's Area Quantification for 5 Patients

	Pre-treatment			Post-treatment		
Patient	C, cm	M, cm	Area, cm^2	C, cm	M, cm	Area, cm^2
1332	3	6	65.00	0	1	9.00
2006	2	3	47.92	0	0.5	5.21
2021	10	11	83.01	0	2	**11.87**
3080	4	6	62.05	0	0	**26.73**
3164	3	4	34.62	0	1	9.01

No ground-truth measurements were available. Evidence for large Barrett's area (>10 sq. cm) post-treatment are indicated in bold (also see Supplementary Figure 3).

Supplementary Table 3. Effects of Challenging Endoscopy Imaging Conditions on Automated Barrett's Length and Area Estimation

	Brightness (Rel.Error)		Blur (Rel.Error)		Uneven Hand Motion (Rel.Error)		Organ Deformation (Rel.Error)		Oblique Camera (Rel.Error)		
Barrett Markers	Mild	Severe[a]	Mild (σ_g=0.2)	Severe (σ_g=0.3)	Mild	Severe[a]	Mild	Severe	Mild	Severe	Normal Case[b] (Rel.Error)
Mb	5.44 (0.17)	3.43 (**0.47**)	5.82 (0.11)	6.19 (0.05)	4.98 (0.24)	4.07 (0.37)	5.11 (0.22)	5.06 (0.23)	6.03 (0.08)	5.54 (0.16)	5.91 (0.10)
C	3.22 (0.37)	3.16 (0.34)	3.68 (**0.57**)	3.50 (0.49)	3.12 (0.33)	2.32 (0.01)	2.78 (0.18)	1.30 (0.44)	2.49 (0.06)	2.48 (0.06)	3.46 (0.47)
Barrett's area 2	35.54 (0.48)	41.09 (0.40)	57.18 (0.17)	49.50 (0.28)	46.79 (0.32)	45.46 (0.34)	64.41 (0.07)	36.54 (0.47)	57.46 (0.17)	32.6 (**0.52**)	70.28 (0.01)

Automated measurements for both mild and severe cases for brightness (dim to dark), simulated blur, random camera motion (ghosting artefacts), organ deformations (external force to mimic insufflation), and oblique camera acquisition (tilted endoscope) are presented with their corresponding relative errors compared with ground-truth measurements on the phantom model (see Supplementary Figure 2*B* and Table 1). Two new videos were acquired for each case (except blur) and tentatively similar camera viewpoints were chosen, including for the normal case. Highest relative error for each Barrett's marker is highlighted in bold. Large error for C in the normal case is solely due to the viewpoint choice.

σ_g, standard deviation used for simulated Gaussian blur on normal frame.

[a]For some cases, generated masks were manually modified.

[b]Standard lighting condition without artefact and deformation.

Supplementary Table 4. Small island measurements

	Island S1		Island S2	
Barrett Islands[a]	Ground Truth, Mean ± SD	Automated, Mean ± SD (Rel.Error)	Ground Truth, Mean ± SD	Automated, Mean ± SD (Rel.Error)
Length, mm	2.3 ± 0.1	2.34 ± 0.33 (0.02)	0.83 ± 0.15	0.64 ± 0.06 (0.23)
Area, mm^2	2.52 ± 0.28	2.91 ± 0.40 (0.15)	NA	0.16 ± 0.04

Automatic measurements for small islands 2 mm (S1) and <1 mm (S2) are presented with their corresponding relative errors compared with ground-truth measurements on the phantom model (see Supplementary Figure 2*B*). Three video frames for each island were used for automated measurements. Vernier calipers (for length) and grid paper (for area) were used to obtain ground truth measurements (also measured 3 times to capture uncertainty in manual measurements).
NA, not available owing to extremely small island area.
[a]Owing to small size, markers were manually placed by zooming and clicking on the image (2 points for length and 3 points for area estimation).
